# Half-Year Longitudinal Seroprevalence of SARS-CoV-2-Antibodies and Rule Compliance in German Hospital Employees

**DOI:** 10.3390/ijerph182010972

**Published:** 2021-10-19

**Authors:** Jonas Herzberg, Tanja Vollmer, Bastian Fischer, Heiko Becher, Ann-Kristin Becker, Hany Sahly, Human Honarpisheh, Salman Yousuf Guraya, Tim Strate, Cornelius Knabbe

**Affiliations:** 1 Department of Surgery, Krankenhaus Reinbek St. Adolf-Stift, Hamburger Strasse 41, 21465 Reinbek, Germany; human.honarpisheh@krankenhaus-reinbek.de (H.H.); tim.strate@krankenhaus-reinbek.de (T.S.); 2 Institut für Laboratoriums- und Transfusionsmedizin, Herz- und Diabeteszentrum Nordrhein-Westfalen, Universitätsklinik der Ruhr-Universität Bochum, Georgstrasse 11, 32545 Bad Oeynhausen, Germany; tvollmer@hdz-nrw.de (T.V.); bfischer@hdz-nrw.de (B.F.); cknabbe@hdz-nrw.de (C.K.); 3 Institute of Medical Biometry and Epidemiology, University Medical Center Hamburg-Eppendorf, Martinistrasse 52, 20246 Hamburg, Germany; h.becher@uke.de; 4 Asklepios Klinik Harburg Abteilung für Psychiatrie und Psychotherapie, Eißendorfer Pferdeweg 52, 21075 Hamburg, Germany; annk.becker@asklepios.com; 5 Labor Lademannbogen MVZ Hamburg, Lademannbogen 61, 22339 Hamburg, Germany; sahly@labor-lademannbogen.de; 6 Clinical Sciences Department, College of Medicine, University of Sharjah, P.O. Box 27272, Sharjah, United Arab Emirates; salmanguraya@gmail.com

**Keywords:** SARS-CoV-2 IgG antibody, health care worker, seroprevalence, COVID-19, health

## Abstract

COVID-19, which is caused by SARS-CoV-2, is an occupational health risk, especially for healthcare employees due to their higher exposure and consequently higher risk of symptomatic and asymptomatic infections. This study was designed to determine the longitudinal seroprevalence of specific immunoglobulin-G (IgG) antibodies in employees in a hospital setting. All employees in a secondary care hospital, including healthcare and non-healthcare workers, were invited to participate in this single-center study. After an initial screening, a 6-month follow-up was carried out, which included serological examination for SARS-CoV-2 IgG antibodies and a questionnaire for self-reported symptoms, self-perception, and thoughts about local and national hygiene and pandemic plans. The seroprevalence of SARS-CoV-2 IgG antibodies was 0.74% among 406 hospital employees (0.75% in healthcare workers, 0.72% in non-healthcare workers), initially recruited in April 2020, in their follow-up blood specimens in October 2020. In this study, 30.54% of the participants reported using the official German coronavirus mobile application and the majority were content with the local and national rules in relation to coronavirus-related restrictions. At the 6-month follow-up, the 0.74% seroprevalence was below the reported seroprevalence of 1.35% in the general German population. The prevalence in healthcare workers in direct patient care compared with that in workers without direct patient contact did not differ significantly. Further follow-up to monitor the seroprevalence in the high-risk healthcare sector during the ongoing global pandemic is essential.

## 1. Introduction

Severe acute respiratory syndrome coronavirus type 2 (SARS-CoV-2), which causes coronavirus disease 2019 (COVID-19), spread from China throughout the whole world beginning in autumn 2019 [1]. With the rising number of infected patients in hospitals, especially in intensive care units, medical staff have become essential cogs in health care systems around the world [2]. Protecting these employees is still one of the most important duties during this crisis, as they are essential to keeping global health care systems intact and functional [3]. As SARS-CoV-2 is transmitted by droplet infection [4], undiagnosed infections in medical staff members can lead to an uncontrolled person-to-person spread to other healthcare workers (HCW) and patients, causing a breakdown of the health care system [5].

In addition to viral detection using polymerase chain reaction (PCR) and rapid antigen tests [6] used in the detection of current infections, serological tests for SARS-CoV-2-specific antibodies are another option, especially for retrospectively detecting asymptomatic or oligosymptomatic infected persons within a defined time period [7]. Studies have shown a high rate of seroconversion for immunoglobulin G (IgG) within two to three weeks after the onset of the disease [8,9,10,11]. The longevity of the specific antibodies is still under discussion [12], and studies have shown no seroconversion for initially PCR-positive tested individuals [13].

The cost-effectiveness and ease-of-use of antibody tests has led to an increasing number of studies describing the seroprevalence of SARS-CoV-2 antibodies in defined groups around the world [14,15,16,17,18,19,20]. Especially among HCW, this tool is frequently used to detect asymptomatic infected individuals, especially following the belief that there potentially exists some protection after a serological immune response.

To evaluate the longitudinal seroprevalence of SARS-CoV-2 in a secondary care mid-sized hospital, a prospective trial was initiated [20]. This evaluation was conducted in an attempt to measure the rule compliance in a highly vulnerable area and evaluate the seroprevalence of SARS-CoV-2 in a secondary care hospital that was involved in the direct patient care during the global pandemic.

## 2. Materials and Methods

The secondary care hospital was located in the province of Schleswig-Holstein near the border of the city of Hamburg. During the entire study period of 6 months (from 14th of April until 20th of October), 36 PCR-confirmed COVID-19 patients were treated in isolation wards and in the intensive care unit at the study center. During the study period, the national protection plan limited private meetings to 10 persons, required face masks to be worn on public transportation, and limited public events. Following social distancing of at least 1.5 m, restaurants and shops remained open.

Within the first study period starting in April 2020 (“Prospective Sero-epidemiological Evaluation of SARS-CoV-2 among Health Care Workers” [20]) all employees between 18 and 90 years—such as cleaning, housekeeping, and administration staff, in addition to HCWs—were given the opportunity to participate in this trial. As in the first phase of the trial, all inhabitants of an affiliated convent were included in the study, as they lived adjacent to the hospital and were at times involved in aspects of non-direct patient care. This enabled a diverse age range of study participants to be included, thereby allowing a good comparison with the general population to be drawn. Written and informed consent was given by all study participants prior to their enrolment.

During the first phase of the trial, all of the participants completed an initial questionnaire with items relating to demographics, general health, and medication, primary working area. and risk of potential SARS-CoV-2 exposure. The participants were asked to provide a weekly oropharyngeal swab and a weekly blood specimen. The results of this phase were published in October 2020 [20].

At the beginning of the study period in April 2020, a strict local hygiene protocol was established that included basic hygiene standards, such as wearing hospital clothing and surgical masks. Personal protective equipment (PPE), including filtering facepiece masks type 2 or 3 (FFP-2/FFP-3), was used routinely by employees working with suspected or confirmed COVID-19 patients. To reduce possible contacts, restrictions for visitors were enforced during this period.

After the first 9-week longitudinal evaluation of the seroprevalence and PCR-positivity within this cohort, a mid-term evaluation of the seroprevalence was performed after 6 months. As in the first phase of the study, no pretesting was performed. The 6-month follow-up presented here also included a questionnaire that evaluated the participants’ thoughts on national hygiene regulatory and travel/social restrictions. This questionnaire also included questions about the participants’ previous SARS-CoV-2 infections, their estimated personal risk for COVID-19, any potential symptoms, and their satisfaction with the local and national protection protocols during October 2020.

At this follow-up after 6 months, all participants were invited a second time to provide a follow-up blood specimen for antibody testing in addition to the questionnaire. The antibody testing was performed using the semiquantitative anti-SARS-CoV-2-ELISA (IgG) from Euroimmun (Lübeck, Germany), which detected the S1 domain of the SARS-CoV-2 spike protein with, according to the manufacturer, a specificity of 99.0% and a sensitivity of 93.8% after day 20 of infection [21]. All positive and equivocally positive results were verified using two different SARS-CoV-2-ELISAs (IgG): one detecting the viral nucleocapsid using the Architect SARS-CoV-2 IgG (Abbott, Wiesbaden, Germany) and the second using the LIAISON SARS-CoV-2 S1/S2 IgG assay (DiaSorin Deutschland GmbH, Dietzenbach, Germany), which detects the S1 and S2 domains of the viral spike protein.

Data were analyzed using IBM SPSS Statistics Version 25 (IBM Co., Armonk, NY, USA). All variables are presented as means or medians with the standard deviation. Categorical variables are shown as numbers with percentages. Fisher’s exact test or chi-square tests were used to determine the relationships between categorical variables depending on the sizes of the groups. Exact 95% confidence intervals were provided where appropriate. The differences between groups were analyzed using *t*-tests and with logistic regression models to adjust for sex and age differences between the groups. A *p*-value < 0.05 was considered statistically significant.

After approval by the Ethics Committee of the Medical Association Schleswig-Holstein, this trial was registered with the German Clinical Trial Register (DRKS00021270). All study activities were conducted in accordance with the Declaration of Helsinki.

## 3. Results

In the initial study period, there were 871 participants. After presenting for a 6-month follow-up, 406 of the initial participants with a median age of 44.18 years returned a completed questionnaire and an additional blood sample for serological testing (follow-up rate: 46.61%). This follow-up cohort included 268 HCW and 138 non-HCW. Of the participants, 76.6% were female (Table 1). The participants included in the follow-up did not differ.

There was a significant difference in the reported presence of typical COVID-19 symptoms between the HCW and non-HCW group, where the HCWs more often presented with the typical symptoms, especially a cough (19.40% vs. 5.07%, *p*-value <0.001). After an adjustment for sex and age in a logistic model, the effect was not found to be significant (*p*-value 0.071). There was no significant correlation between the typical symptoms and a positive PCR test (6 out of 159 participants with the symptoms, *p*-value 0.061) and between the symptoms and seroconversion (2 out of 159 participants, *p*-value 0.564). Even potentially highly specific symptoms such as anosmia or taste distortion (one out of five participants reported this symptom, *p*-value 0.095) showed no significant correlation with a positive PCR test within the study population.

The overall rate of the self-reported ever-PCR-positive participants was 1.97%. The rates did not differ between the groups (1.87% in HCW vs. 2.17% in the non-HCWs). The rate of positive anti-SARS-CoV-2 IgG antibodies was 0.74% in the total group. The percentages also did not differ between the groups (0.75% vs. 0.72% in the non-HCWs).

Eight participants (1.97%) reported having a previous positive SARS-CoV-2-PCR test, while only three participants showed a positive result for anti-SARS-CoV-2 IgG antibodies at the 6-month follow-up. Following this evaluation, six participants with a previous positive SARS-CoV-2-PCR test did not show a positive antibody test. One participant with a positive antibody result reported no previous positive PCR test result (Table 2).

Not all participants reporting a previous positive PCR result reported any kinds of symptoms, whereas two of the three participants with a positive antibody status reported distinct symptoms (Table 2).

In the non-HCW follow-up cohort, the majority listed prior comorbidities, with a significant difference seen compared to the HCW group (40.67% in the HCWs, 52.17% in the non-HCWs, *p*-value 0.027) in the univariate analysis. The area of cardiac comorbidities differed significantly between both groups (14.55% vs. 23.91% in the non-HCWs, *p*-value 0.019). The effect remained significant in the logistic regression analysis (*p*-value 0.012). The difference in the body mass index between both groups was also significant (*p*-value < 0.001). There was no significant difference in smoking behaviors between both groups. In a logistic model adjusted for sex, age, BMI, and smoking, the difference between the groups disappeared.

In the HCW, the usage of the official mobile warning application was significantly higher than in the non-HCW group (34.33% vs. 23.19% in the non-HCWs, *p*-value 0.023 after adjusting for age and sex in a logistic model).

The personal risk was estimated to be low or lower in 68.7% of all participants without significant differences seen between both groups (68.8% in the non-HCWs, 68.7% in the HCWs).

The rates of participants requiring a period of quarantine did not differ significantly between the HCWs and non-HCWs. The main reason for requiring quarantine in the HCWs was due to professional contact with positive tested patients or co-workers (22/34), whereas the reasons for isolation in the non-HCWs were distributed between professional contact (7/14), private contact (2/14), and returning from holidays in high-risk regions (5/14).

**Figure 1:**
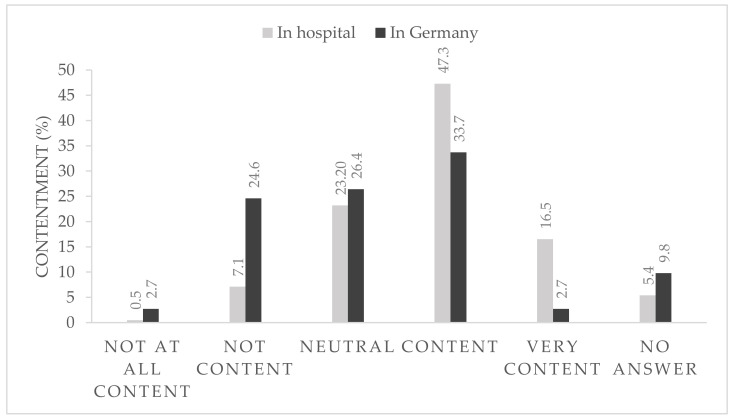
Contentment (%) with local (**grey**) and national (**black**) pandemic protocol, *n* = 406.

After 6 months of strict hygiene protocols within the hospital and with the rapidly changing national pandemic regulations, 36.4% of the participants were content or very content with the national regulations (Figure 1) and 63.8% reported that they were very content or at least content with the local in-hospital protocols (Figure 1).

**Figure 2:**
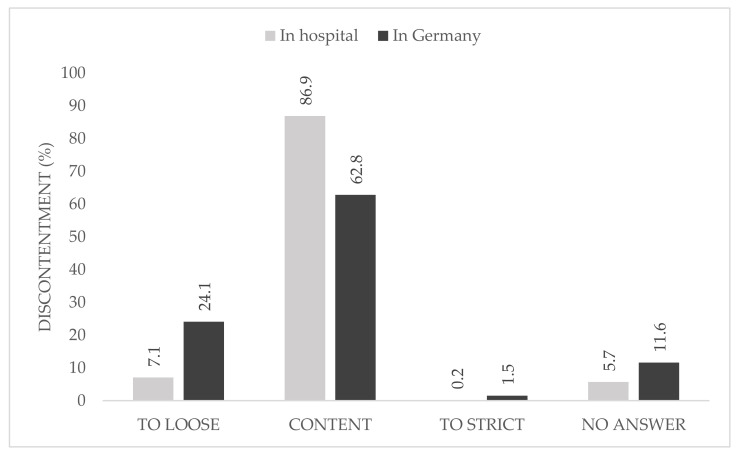
Causes for discontentment (%) with local (**grey**) and national (**black**) pandemic protocol, *n* = 406. Neutral, content, and very content are summarized under “content” in this figure.

When asked about the causes of their discontent with the national regulations, 24.1% described them as too loose, whereas only 1.5% declared the restrictions in Germany to be too strict. At local hospital level, one participant described the rules as too strict (Figure 2).

**Figure 3:**
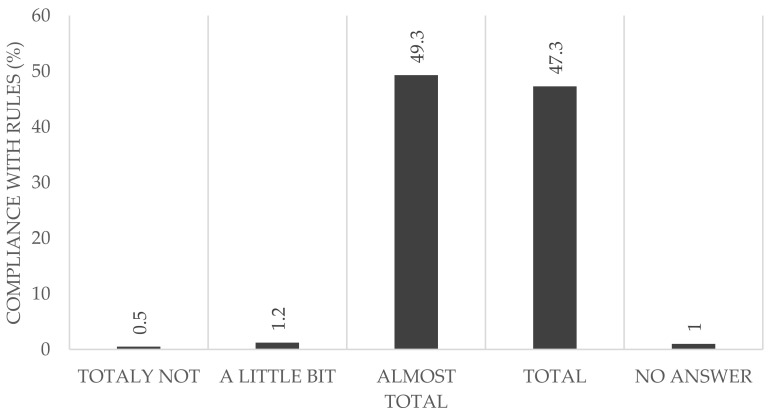
Employees self-reporting compliance with rules (%), *n* = 406.

Almost half of the participants reported that they adhered to the rules completely (HCWs: 42.2%; non-HCWs: 57.2%) (Figure 3). Considering all of the participants reporting that they followed the rules almost completely (HCWs: 54.1%, non-HCWs: 39.9%), we found no difference between both groups (96.3% in the HCW vs. 97.1% in the non-HCWs).

## 4. Discussion

This study provides one of the first 6-month follow-up reports on the occurrence of specific anti-SARS-CoV-2 IgG antibodies within a high-risk group of hospital employees caring for COVID-19 patients. Within the follow-up period, three participants (0.74%) showed seroconversion. Eight participants (1.97%) reportedly had a positive throat swab prior to their blood sample being taken.

### 4.1. Seroprevalence

In this trial, not all participants with a reported positive PCR showed specific antibodies in the follow-up blood specimen. This could have been caused by an early decrease in their antibody titer, as Ibarrondo et al. and Long et al. have shown [12,22]. Furthermore, different studies have suggested that asymptomatic or oligosymptomatic infected patients do not seroconvert [21,23,24]. Tan et al. have shown that patients with a higher disease severity are more likely to develop a stronger antibody response, as we saw in two participants with a high IgG-ratio in our study (Table 2) [25].

The overall seroprevalence of anti-SARS-CoV-2 IgG antibodies in this trial is comparable to data from other German hospitals, ranging from 0% in a 5-day setting [14] up to >12% in a larger longitudinal study (as reported by Malfertheimer et al. [16]). The longitudinal data reporting the conversion to seropositive status are limited so far. Behrens et al. showed in their CoCo trial a longitudinal prevalence of anti-SARS-CoV-2 IgG-antibodies of 1.86% within 6 weeks [26]. By comparison, in our initial data from the initial 9-week phase of the study we observed a seroprevalence of 4.36% (with the limitation that different assays were used in that evaluation) [20]. Our current data are comparable with the seroprevalence found in the normal German population of around 0.91% between March and May 2020 [27]. These rates are significantly lower than data reported by a population-based trial from Switzerland, which found a seroprevalence up to 10.6% during the same time period [28]. According to the large seroprevalence study conducted by the Robert Koch Institute in blood donors, the seroprevalence within the German population was found to be 1.35% in almost 50,000 tested blood samples [29].

As the seroprevalence correlates with the local infection rate, it is difficult to compare data from other regions around the world. Table 3 provides an overview of seroprevalence studies carried out on health care providers in European countries.

Due to limitations in our study, we were unable to assess possible infection routes and thus could not evaluate whether the prior infections were work-acquired or due to community exposure to SARS-CoV-2. Paderno et al. have shown infection routes in HCWs [35].

### 4.2. Self-Perception and Evaluation

Almost 40% of all participants reported symptoms suggesting potential SARS-CoV-2 infection, such as fever or cough. Due to the low PCR positivity rate and seroconversion, there is no significant correlation between these symptoms and possible SARS-CoV-2 infection.

In contrast to the research presented by Behrens et al., the majority of the participants in this study estimated their own personal risk of a prior infection to be low or very low, independent of their working area [7].

In the cohort presented here, 30.54% of the participants used the official mobile application “Corona-Warn-App” presented by the Robert Koch Institute [36]. There was a significantly higher user rate in the HCW group. This might be due to the younger mean age of this group. As this application uses a decentralized system without collecting data, the only known data are the number of downloads instead of the number of actual users. According to the federal German government, the application was downloaded 20 million times by the 20th of October 2020 [37]. Comparing this with the number of smartphone users in Germany, which was 58 million in 2019 [38], the user rate across society seems to be around 34%, which is comparable with the rates found in our study individuals.

Looking at the rate of quarantine for different reasons, 10.8% is a relevant proportion for a healthcare provider (12.31% in the HCWs vs. 9.42 in the non-HCWs). This, in combination with the high rate of employees presenting specific symptoms, leads us to the conclusion that a protocol for screening that uses PCR or rapid antigen tests is needed in order to prevent the breakdown of a healthcare system during a global pandemic [39].

In addition to the serological follow-up examination, the questionnaire included questions about people’s perception of the local and global pandemic protocols. The data showed that the majority of study participants abided by the rules almost completely or else completely, without significant differences seen between the two groups (non-HCW: 97.1%, HCW: 96.2%). Only a few participants declared that they were unable to abide by the rules. This is an important factor, as the behavior of even a few individuals could jeopardize the whole hospital and in addition the whole health care system.

The majority of the participants were content with the local hygiene protocols and pandemic plan and no participant described it as too loose, whereas a quarter were not content with the national hygiene protocols and pandemic plans. In this group, just 1.5% rated the national rules as too strict.

### 4.3. Limitation

The major limitation of this study is its single-center design. Furthermore, the participation rate of 46.66% of all initially included participants in the follow-up is relatively low. This might be due the effects of shift work, holidays, or participants no longer working in the study center, as well as due to loss in the follow-up. Females are highly overrepresented in both groups, representing a common trend in health care workers [40]. As we refer to reported PCR positivity and not to results of a test performed within this study, false-positive results might skew our research findings. This could explain the low antibody level seen in some of the participants with positive PCR results. However, different studies describe a missing seroconversion in participants with no or few symptoms, as was mentioned above. Due to the low number of seroconverted participants within this 6-month follow-up, this study has too low a power for it to detect the characteristics of seroconverted participants and encourage such a seroconversion. The only possibility for grouping the participants is according to their profession and not their working area, as a large number of employees work in multiple areas of the hospital (in both high- and low-risk areas). This limitation may be compounded by the fact that in the study center there were no completely separated pathways for possible COVID-19 patients. Additional testing for other antibodies such as IgM and IgA was not performed in this study in order to retain its comparability with the initial phase of this trial. Such additional testing might provide further information, particularly about recent infections or seronegative participants with reported PCR positivity. The use of additional tests, such as for the cellular immune response, might be useful in further studies.

## 5. Conclusions

The data presented in this study provide one of the first longitudinal sero-epidemiological assessments of the prevalence of specific antibodies against SARS-CoV-2 in healthcare workers compared with hospital employees not directly caring for patients.

Moreover, this study presents the self-perception of hospital employees with regard to their views on the local and national protection protocols—that is, the views of individuals intimately involved in the fight against this global pandemic.

## Figures and Tables

**Table 1:** Comparison of the characteristics of enrolled hospital employees by working area (healthcare vs. non-healthcare).

Characteristics	HCW (*n* = 268)	Non-HCW (*n* = 138)	*p*-Value
Median age (mean ± SD)	41.38 ± 12.09	49.66 ± 15.17	**<0.001 ^c^**
Female sex, *n* (%)	191 (71.27)	120 (86.96)	**<0.001 ^a^**
BMI (mean ± SD)	25.80 ± 5.71	26.11 ± 6.87	0.624 ^c^
Chronic medical condition, *n* (%)	109 (40.67)	72 (52.17)	**0.027 ^a^**
Cardiac	39 (14.55)	33 (23.91)	**0.019 ^a^**
Pulmonary	23 (8.58)	15 (10.87)	0.454 ^b^
Metabolic	40 (14.93)	23 (16.67)	0.646 ^a^
Immunologic	14 (5.22)	7 (5.07)	1.000 ^b^
Other	42 (15.67)	26 (18.84)	0.418 ^a^
Current smoker, *n* (%)	70 (26.12)	28 (20.29)	0.194 ^a^
Specific symptoms, *n* (%)	119 (44.40)	40 (28.99)	**0.003 ^a^**
Cough	52 (19.40)	7 (5.07)	**<0.0001 ^a^**
Fever	19 (7.09)	4 (2.90)	0.112 ^b^
Sore throat	66 (24.63)	23 (16.67)	0.066 ^a^
Sniff	73 (27.24)	29 (21.01)	0.171 ^a^
Headaches	56 (20.90)	24 (17.39)	0.400 ^a^
Fatigue	49 (18.28)	15 (10.87)	0.052 ^a^
Taste distortion	3 (1.12)	2 (1.45)	1.000 ^b^
Anosmia	3 (1.12)	2 (1.45)	1.000 ^b^
Use of official warning mobile application, *n* (%)	92 (34.33)	32 (23.19)	**0.021 ^a^**
Quarantine, *n* (%)	33 (12.31)	13 (9.42)	0.414 ^b^
Reported positive PCR test, *n* (%)	5 (1.87)	3 (2.17)	1.000 ^b^
Positive anti-SARS-CoV-2 IgG antibodies	2 (0.75)	1 (0.72)	1.000 ^b^

^a^ Chi-square test; ^b^ Fisher’s exact test; ^c^ *t*-Test; SD: standard deviation; BMI: body mass index; PCR: polymerase chain reaction.

**Table 2:** Correlation of positive PCR test, anti-SARS-CoV-2 IgG antibodies and symptoms. Euroimmun assay (equivocal: ratio ≥ 0.8 to <1.1, seropositive: ratio ≥ 1.1).

Participant	Reported Positive PCR	Initial Euroimmun IgG Ratio	Follow-Up Euroimmun IgG Ratio	Reported Symptoms
1	Yes	3.4	2.9	Long-term cough, headaches, fatigue
2	Yes	0.1	0.1	No
3	Yes	0.2	0.4	No
4	Yes	0.1	0.2	Fever, fatigue, sore throat, limb pain
5	Yes	0.2	0.2	Fatigue, sore throat, limb pain
6	No	0.6	1.2	No
7	Yes	0.1	0.1	Cough, headaches, fatigue
8	Yes	1.3	1.0	Fever
9	Yes	-	3.2	Headaches, fatigue, sore throat, limb pain, taste distortion, anosmia

**Table 3:** Seroprevalence of anti-SARS-CoV-2 IgG antibodies in different European countries.

Study	Country	Design	Participants	Seroprevalence of Anti-SARS-CoV-2IgG Antibodies
▪ Krankenhaus Reinbek	Germany	single-center	406	0.74%
▪ Steensels et al. [30]	Belgium	single-center	3056	10.6%
▪ Garcia-Basteiro et al. [31]	Spain	single-center	578	9.3%
▪ Jespersen et al. [32]	Denmark	multi-center	17,971	3.4%
▪ Sotgiu et al. [33]	Italy	single-center	202	14.4%
▪ Rudberg et al. [34]	Sweden	single-center	2149	19.1%

## Data Availability

The data presented in this study are available on request from the corresponding author. The data are not publicly available due to privacy reasons.
